# Highly efficient dual-level grating couplers for silicon nitride photonics

**DOI:** 10.1038/s41598-022-19352-9

**Published:** 2022-09-14

**Authors:** Valerio Vitali, Cosimo Lacava, Thalía Domínguez Bucio, Frederic Y. Gardes, Periklis Petropoulos

**Affiliations:** 1 Optoelectronics Research Centre, Highfield Campus, University of Southampton, Southampton, SO17 1BJ UK; 2 Electrical, Computer and Biomedical Engineering Department, University of Pavia, Pavia, 27100 Italy

**Keywords:** Integrated optics, Silicon photonics

## Abstract

We propose and numerically demonstrate a versatile strategy that allows designing highly efficient dual-level grating couplers in different silicon nitride-based photonic platforms. The proposed technique, which can generally be applied to an arbitrary silicon nitride film thickness, is based on the simultaneous optimization of two grating coupler levels to obtain high directionality and grating-fibre mode matching at the same time. This is achieved thanks to the use of two different linear apodizations, with opposite signs, applied to the two grating levels, whose design parameters are determined by using a particle swarm optimization method. Numerical simulations were carried out considering different silicon nitride platforms with 150, 300, 400 and 500 nm thicknesses and initially employing silicon as the material for the top level grating coupler. The use of Si-rich silicon nitride with a refractive index in the range 2.7–3.3 for the top layer material enabled to obtain similar performance (coupling efficiency exceeding − 0.45 dB for the 400 nm thick silicon nitride platform) with relaxed fabrication tolerances. To the best of our knowledge, these numerical results represent the best performance ever reported in the literature for silicon nitride grating couplers without the use of any back-reflector.

## Introduction

Over the last two decades, silicon photonics has revolutionised a wide range of research fields including all-optical processing^1,2^, quantum technologies^3,4^ and sensing^5,6^. The success of the Silicon-On-Insulator (SOI) platform can be mainly attributed to its CMOS compatibility and to the high index contrast between the Si core (n$_{Si}$ = 3.48 at 1550 nm) and the SiO$_2$ cladding (n$_{SiO_2}$ = 1.44 at 1550 nm), which allows a strong optical confinement and small bending radius. However, some of the properties of Si compromise the development of efficient devices for the aforementioned applications. For example, its large thermo-optic coefficient makes the behaviour of SOI devices strongly dependent on temperature variations^7^. Moreover, SOI devices suffer from the high Two-Photon Absorption (TPA) coefficient of Si at telecommunication wavelengths^8^, which hinders the use of these systems for high-power density and all-optical processing applications. For these reasons, silicon nitride (Si$_3$N$_4$) has attracted significant attention for the realization of integrated photonic devices, both for linear and nonlinear applications^9,10^. Si$_3$N$_4$ exhibits negligible TPA in the infrared spectrum, its thermo-optic coefficient is one order of magnitude lower than that of Si, which results in greater temperature tolerance^11,12^, and its transparency window reaches wavelengths as low as 500 nm, enabling sensing applications in the visible.

The lower refractive index contrast between Si$_3$N$_4$ (n$_{Si_3N_4}$ = 2 at 1550 nm) and SiO$_{2}$, compared to the case of SOI devices, enables lower propagation losses to be achieved and gives rise to greater tolerances in the fabrication. However, one downside is that the realization of efficient grating couplers (GCs), to couple light beams to and from an out-of-plane optical fibre, is more challenging than in the case of Si waveguides. This is because the relatively low refractive index of Si$_3$N$_4$ results in an increased grating period and, therefore, a smaller number of scattering elements for a given optical fibre mode diameter. In addition, the relatively low index contrast between Si$_3$N$_4$ and SiO$_{2}$ restricts the scattering strength of the individual periods of the grating, making it particularly challenging to match the optical intensity profile radiated by a GC with the Gaussian profile of a standard optical fibre. As reported in the literature^13,14^, single-level partially-etched Si$_3$N$_4$ GCs require a waveguide thickness > 800 nm to achieve > 80% directionality (defined as the percentage of the power incident on the GC that is scattered upwards towards the optical fibre). Several approaches were reported to increase the directionality and, hence, the coupling efficiency (CE) for lower Si$_3$N$_4$ waveguide thicknesses. One of the most common approaches makes use of metal reflectors^15^ or Distributed Bragg Reflectors (DBRs)^16–18^ to recover the light scattered towards the substrate. A reflectivity higher than 92% was numerically demonstrated using a bottom Si grating reflector, with a resulting simulated CE of − 0.88 dB for an apodized 400 nm-thick Si$_3$N$_4$ GC^19^. CE values greater than 90% (− 0.45 dB) were numerically simulated by combining a bottom DBR and a chirp generator algorithm^18^, with an experimentally measured CE of − 1.17 dB at 1571 nm for an air-cladding, partially etched GC on a 500 nm thick Si$_3$N$_4$ platform. Another way to increase directionality is to employ a double-etched grating design. For example, a two-step staircase-shaped grating profile was used to produce a blazing effect in a 600 nm Si$_3$N$_4$ waveguide, allowing to achieve a simulated and experimental CE of − 0.66 dB and − 1.5 dB, respectively^20,21^. A third approach is the realization of dual level GCs, either by hybridizing a Si$_3$N$_4$ grating with a Si grating underneath^13,22,23^ or by considering a Si$_3$N$_4$–Si$_3$N$_4$ bi-layer GC^14^. Simulated and experimental CEs of − 1 dB and − 1.3 dB, respectively, were reported for such a Si$_3$N$_4$-SOI platform for a 400 nm thick Si$_3$N$_4$ waveguide^13^. Table 1 summarises the numerical and experimental results reported in the literature for different GCs demonstrated on various Si$_3$N$_4$ platforms in the S–C–L wavelength bands. For convenient comparison, we have also included in the table the numerical results that will be presented in this work. Overall, GCs with embedded back-reflectors still outperform the other proposed approaches. However, this solution presents some limitations when it comes to fabrication. For example, the realization of metal back-reflectors may require the use of non-CMOS compatible materials that can be difficult to insert in a metal-free CMOS fabrication environment. Conversely, DBRs can be realized using a stack of several amorphous Si^24^ or Si$_3$N$_4$ layers^16^, but this requires extra-processing steps to define the bottom reflector and may result in poor fabrication tolerances and a significant deviation from the simulated values. Another issue is the resulting Si$_3$N$_4$-film stress due to the deposition of the layers underneath, with the possible formation of cracks and an increase in the propagation losses. For these reasons, solutions that do not require the use of back-reflectors are generally preferred.

**Table 1:** Comparison of different GCs for the S-C-L bands demonstrated on various Si$_3$N$_4$ platforms.

Si$_3$N$_4$ Height (nm)	Simulations		Experiments		Notes	Ref
	CE (dB)	1 dB-BW (nm)	CE (dB)	1 dB-BW (nm)		
100	− 3.8	–	− 5	75 (3dB-BW)	Amorphous-Si-on-Si$_3$N$_4$ dual-level GC	^25^
220	− 2.28	57.7	− 2.56	46.9	Apodized bilayer GC	^14^
300	− 0.48	45	–	–	Bottom DBR+chirp generator algorithm	^18^
325	− 1	54	− 1.75	76.34 (3dB-BW)	Multilayer reflector+apodized GC	^16^
325	− 1.3	–	− 4.5	68 (3dB-BW)	Bottom DBR (10 layers)	^26^
400	− 0.88	70	–	–	Apodized GC+bottom Si grating reflector	^19^
400	− 1	82	− 1.3	80	Si$_3$N$_4$-on-SOI dual-level GC	^13^
400	− 3.9	67	− 4.2	67	Fully-etched trenches	^27^
400	− 1.2	–	− 2.6	53	Bottom DBR	^17^
400	− 2.32	102	− 2.5	53	Bottom DBR	^28^
400	− 0.38	42	− 1.24	39	Bottom DBR+chirp generator algorithm	^18^
400	− 1.13	75	− 2.58	52	Bottom DBR	^24^
400	− 2.52	–	− 5.1	60	DUV-lithography (500nm resolution)	^29^
500	− 0.5	33	− 1.17	40	Bottom DBR+chirp generator algorithm	^18^
500	− 1.34	56	− 2.29	49	Bottom DBR	^24^
600	− 0.66	22.3	− 1.5	60 (3dB-BW)	Two-step staircase-shaped GC	^20,21^
600	− 2.13	63	− 2.5	65	Si$_3$N$_4$-on-SOI dual-level GC	^23^
700	− 2.8	–	− 3.7	54	One partial etching step	^30^
150	− 0.75	57	–	–	Dual level Si–Si$_3$N$_4$ GC (this work)	–
300	− 0.7	31	–	–	Dual level Si–Si$_3$N$_4$ GC (this work)	–
400	− 0.39	28	–	–	Dual level Si–Si$_3$N$_4$ GC (this work)	–
500	− 0.39	21	–	–	Dual level Si–Si$_3$N$_4$ GC (this work)	–

In this paper, we propose and numerically demonstrate a method for the design of dual-level GCs for silicon nitride photonics that allows achieving CE greater than − 0.4 dB and relaxed fabrication tolerances. The GC layout consists of a bottom Si$_3$N$_4$ guiding layer and a top layer with refractive index n$_{Si_3N_4}$ $\le$ n $\le$ n$_{Si}$, separated by an additional thin SiO$_2$ spacer. In the proposed approach, the parameters of the two levels are optimized simultaneously by means of two linear apodizations with opposite signs, which are applied to the two GCs, whose final behavior can be considered equivalent to that of a single GC with a combination of two levels of teeth. Unlike previously proposed bi-layer configurations, where only stoichiometric Si$_3$N$_4$ and Si were considered, we also investigated, for the first time, the use of Si-rich silicon nitride (Si$_x$N$_y$), whose refractive index can be precisely tuned to take any value between those of stoichiometric Si$_3$N$_4$ and Si by varying the gas composition of the film forming reactants during the deposition process^31^. This provides an additional free parameter, enabling designs that are more robust to fabrication imperfections through the use of a top Si$_x$N$_y$ GC layer with an intermediate refractive index. Thanks to its versatility, the proposed design methodology can be applied in principle to any Si$_3$N$_4$ photonic platform with an arbitrary waveguide thickness.

## Layout and design of dual-level grating couplers

The amount of power that can be coupled from a GC to an out-of-plane optical fibre, i.e. its CE, can be generally expressed by the following equation:

1 $$\begin{aligned} CE [dB] = 10 \cdot log_{10}((1-R) \cdot D \cdot FM) \end{aligned}$$

where *R* is the reflectivity (which accounts for the power that is reflected back into the optical waveguide), *D* is the directionality and *FM* is the field matching between the field scattered by the GC and the Gaussian power distribution of the optical fibre mode. Large CE values can only be achieved by having large *D* and *FM* and low reflectivity *R*, at the same time. In standard uniform GCs, trenches with a constant length $L_e$ and fixed depth *e* are etched in the Si$_3$N$_4$ waveguide with a periodicity $\Lambda$. By defining the GC fill-factor *F* as the ratio between the length of the tooth (the un-etched section) $L_o$ over the total length $\Lambda$ of the scattering element, the effective refractive index $n_{eff}$ of the GC can be defined as:

2 $$\begin{aligned} n_{eff} = F \cdot n_{o} + (1 - F) \cdot n_{e} \end{aligned}$$

where $n_o$ and $n_e$ stand for the effective indices of the original Si$_3$N$_4$ slab and the etched regions, respectively. The periodic change in the effective refractive index between the teeth and trenches of the Si$_3$N$_4$ waveguide results in the diffraction of the optical mode to free space at a certain angle^32^. The GC period can be calculated by using the Bragg law: in a GC radiating (out of plane) at a certain angle $\theta$, to achieve constructive interference, the phase delay $\phi _{in-plane}$ experienced by the wave propagating in-plane between two adjacent scatterers and the phase delay $\phi _{out-of-plane}$ acquired by the wave diffracted upwards by the first scatterer have to exhibit a difference of $2 \pi$ . This can be expressed by using the following equations^33,34^:

3 $$\begin{aligned}&\phi _{in-plane} = \phi _{out-of-plane} + 2\pi \end{aligned}$$

4 $$\begin{aligned}&k_0n_{eff}\Lambda = k_0 n_{air} (\Lambda sin(\theta )) + 2\pi \end{aligned}$$

where $k_0$ represents the vacuum wavenumber, $n_{air}$ is the refractive index of the air, $\Lambda$ is the GC period (radiative unit length) and $\theta$ is the angle of radiation in the air. The GC period $\Lambda$ can therefore be calculated as:

5 $$\begin{aligned} \Lambda =\frac{\lambda _c}{(n_{eff} - sin(\theta ))} \end{aligned}$$

where $\lambda _c$ is the coupling wavelength. If a uniform GC is considered, the scattered field profile follows an exponential decay and the radiated optical power *P*(*z*) can be expressed as follows^35^:

6 $$\begin{aligned} P(z) = P(0) e^{-2 \alpha z} \end{aligned}$$

where $\alpha$ is referred to as the grating coupling strength of each scatterer (that is constant over the whole grating surface for a uniform GC) and *z* is the direction of light propagation along the GC length. The modal overlap between *P*(*z*) and the optical fibre power distribution (Gaussian) is therefore limited, and the CE is significantly reduced. As already reported in the literature, a linear apodization of the GC can significantly increase the CE^32,36^. In this case, the parameter $\alpha$ has to be modified to allow for a coupling strength variation along the GC length. This can be expressed by the following equation^33,35^:

7 $$\begin{aligned} \alpha (z) = \frac{1}{2}\frac{G^2(z)}{1-\int _{0}^z G^2(h)dh} \end{aligned}$$

where *G*(*z*) represents the normalized fibre Gaussian distribution.

The adoption of a linear apodization with a linear variation of the *F* parameter along the GC length has two positive effects: the amount of optical power scattered by the first few periods is reduced, allowing to achieve a Gaussian-like radiated power distribution, and the optical impedance matching between the un-etched waveguide and the GC is improved, with a consequent reduction in the reflectivity.

**Figure 1:**
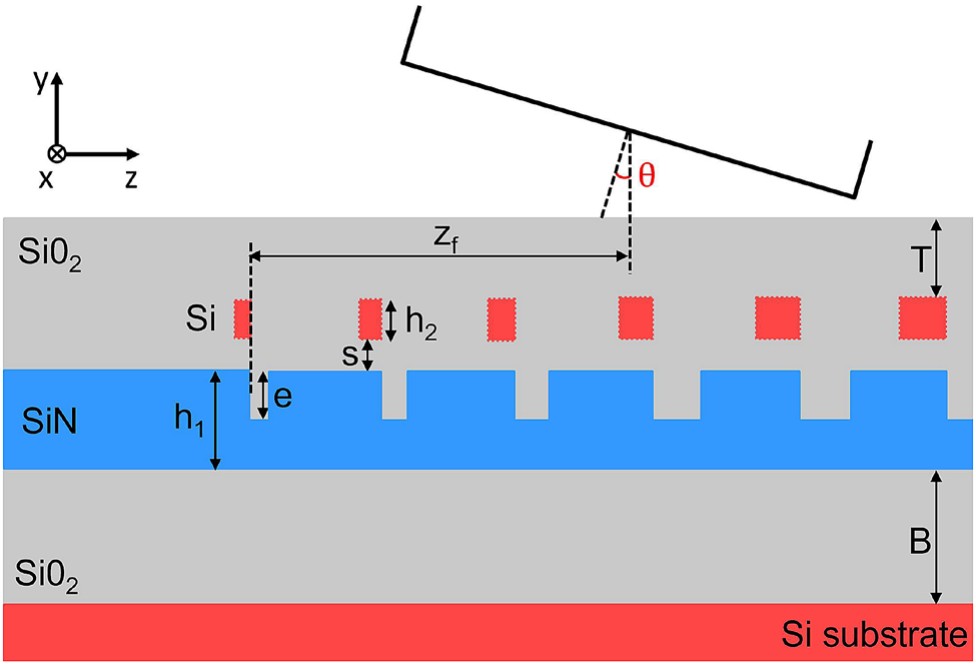
2D schematic view of the proposed dual-level Si–Si$_3$N$_4$ GC.

Based on these considerations, the layout of the proposed dual-level Si–Si$_3$N$_4$ GC is shown in Fig. 1 and consists of two GC layers separated by a thin SiO$_2$ spacer with thickness *s*. The bottom Si$_3$N$_4$ waveguide has a fixed thickness $h_1$, whose value is chosen according to the specific application, and an etching depth *e*, while the top Si GC has a thickness $h_2$ and is fully etched. In the second part of the paper, a Si$_x$N$_y$ top level material is considered, with a refractive index value that varies, ranging from that of Si$_3$N$_4$ to the one of Si. The two GC layers have the same period $\Lambda$, with their teeth aligned on the furthermost border, and their fill-factors are defined by two linear apodization functions, with opposite signs. The equation used to apodize the bottom GC is the following:

8 $$\begin{aligned} F_1 = F_{in,1} - R_1 \cdot z \end{aligned}$$

where $F_{in,1}$ is the initial fill-factor of the first bottom scattering element, $R_1$ is the bottom linear apodization factor and *z* is the distance of each scattering element from the starting position of the GC. Similarly, the expression defining the apodization of the top layer is:

9 $$\begin{aligned} F_2 = F_{in,2} + R_2 \cdot z \end{aligned}$$

where $F_{in,2}$ is the initial fill-factor of the first top scattering element and $R_2$ is the top linear apodization factor. An extra top tooth is included before the first GC period to ensure that the coupling between the bottom and top layers at the beginning of the GC is weak and increase the CE, as was already proven in^13,22^.

The use of inverse linear apodizations for the two layers has two main advantages: the first is that it allows the back-reflection at the GC interface and the mismatch between the Si$_3$N$_4$ optical mode and the composite dual-level GC mode to be minimized, thanks to the gradual decrease of the bottom fill-factor and the increase of the top fill-factor. The second advantage is that it reduces the set of simulation parameters: considering that the initial fill-factor values $F_{in,1}$ and $F_{in,2}$ are constrained by the minimum feature size achievable in the fabrication process, the GC tooth and trench dimensions are dictated only by three parameters, namely $R_1$, $R_2$ and the period $\Lambda$. This allows the simultaneous optimization of both the bottom and top GC dimensions together with the other free GC parameters, i.e. *e*, $h_2$, *s* and the thickness of the top SiO$_2$ cladding *T*. A standard single-mode fibre (SMF-28) for telecom and datacom photonic applications was considered, with an outer diameter of 125 $\upmu$m and a mode field diameter (MFD) of 10.4 $\upmu$m. The fibre parameters to be optimized are the coupling angle $\theta$ and the fibre offset $z_f$, that is the distance between the start of the GC and the centre of the fibre. With these considerations in place, a GC design strategy was devised. This was carried out in three steps, as will be presented in the following section.

## Design methodology and optimisation

### Dual-level Si–Si$_3$N$_4$ grating couplers

In order to present the optimization procedure and compare the achievable results with those reported in the literature, the waveguide layer thickness $h_1$ was selected and fixed at 400 nm, since this value matches one of the most widely adopted Si$_3$N$_4$-based photonic platform configurations and has already been used in several GC demonstrations reported in the literature^13,17–19,24,27,37^. The thickness of the bottom silica layer (BOX) *B* was initially considered as a fixed parameter and set to 3 $\upmu$m to minimize the power coupling from the Si$_3$N$_4$ waveguide to the Si substrate. The use of a top level Si GC to increase the CE was investigated in the first stage, with the GC layout shown in Fig. 1. The set of the GC free parameters is {$e, h_2, R_1, R_2, \Lambda , s, T, \theta , z_f$}. We considered a minimum value of 20 nm for the SiO$_2$ thickness *s*, which can be achieved in fabrication by performing chemical mechanical polishing (CMP) after a SiO$_2$ layer deposition^38^. Regarding the values of the initial fill-factors, it was previously reported that increasing the initial fill-factor (and, hence, reducing the dimension of the first trench) in a single-level GC results in an increase in CE^39^. In our design, the maximum value of the bottom initial fill-factor $F_{in,1}$ is limited by the dimension of the first bottom trench, while the minimum value of the top initial fill-factor $F_{in,2}$ is constrained by the dimension of the first top tooth. These dimensions cannot be smaller than the minimum feature size given by the chosen fabrication process. We set a minimum feature size of 100 nm, which is compatible with scalable deep UV lithographic systems. As an example, considering a GC period of 1 $\upmu$m, $F_{in,1}$ yields a value of 0.9, while $F_{in,2}$ is equal to 0.1. An additional tooth was added at the start of the GC in the top layer, following the study developed in^13,22^. We numerically found that the width of this additional tooth, which maximized the CE in our design, corresponded to the one given by the minimum feature size of 100 nm, in line with previous studies^39^. This allowed the mode-mismatch and back-reflection at the dual-level grating interface to be minimized.

**Figure 2:**
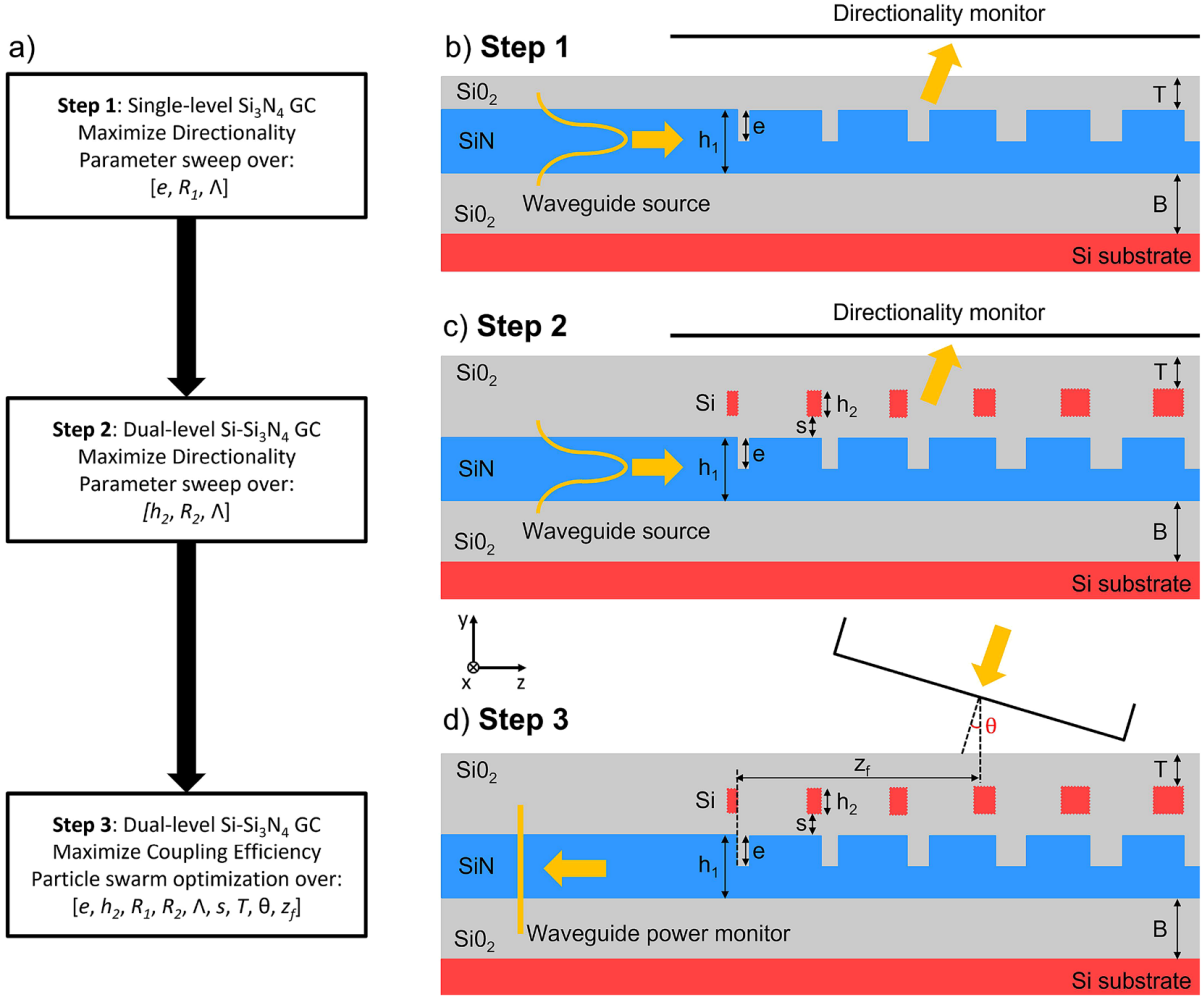
(**a**) Schematic of the optimization strategy followed in the design of the dual-level Si–Si$_3$N$_4$ GC; cross-sectional schematic and simulation layout used to maximize the directionality in (**b**) step 1 and (**c**) step 2 and the CE in (**d**) step 3 of the design process.

The GC design was carried out in three steps, which are summarized in the diagram of Fig. 2a and are represented schematically in Fig. 2b–d. Two parameter sweeps were initially performed to study the GC directionality: the first one considered just the single Si$_3$N$_4$ layer, while the second Si layer was added on in the second step. In the third step, a particle swarm optimization algorithm was applied to maximize the CE. This accounts both for the directionality and the GC-optical fibre field matching. It should be noted that the two parameter sweeps were only performed to roughly find the initial starting point for the particle swarm optimization, which represents the core of the optimization strategy.

**Figure 3:**
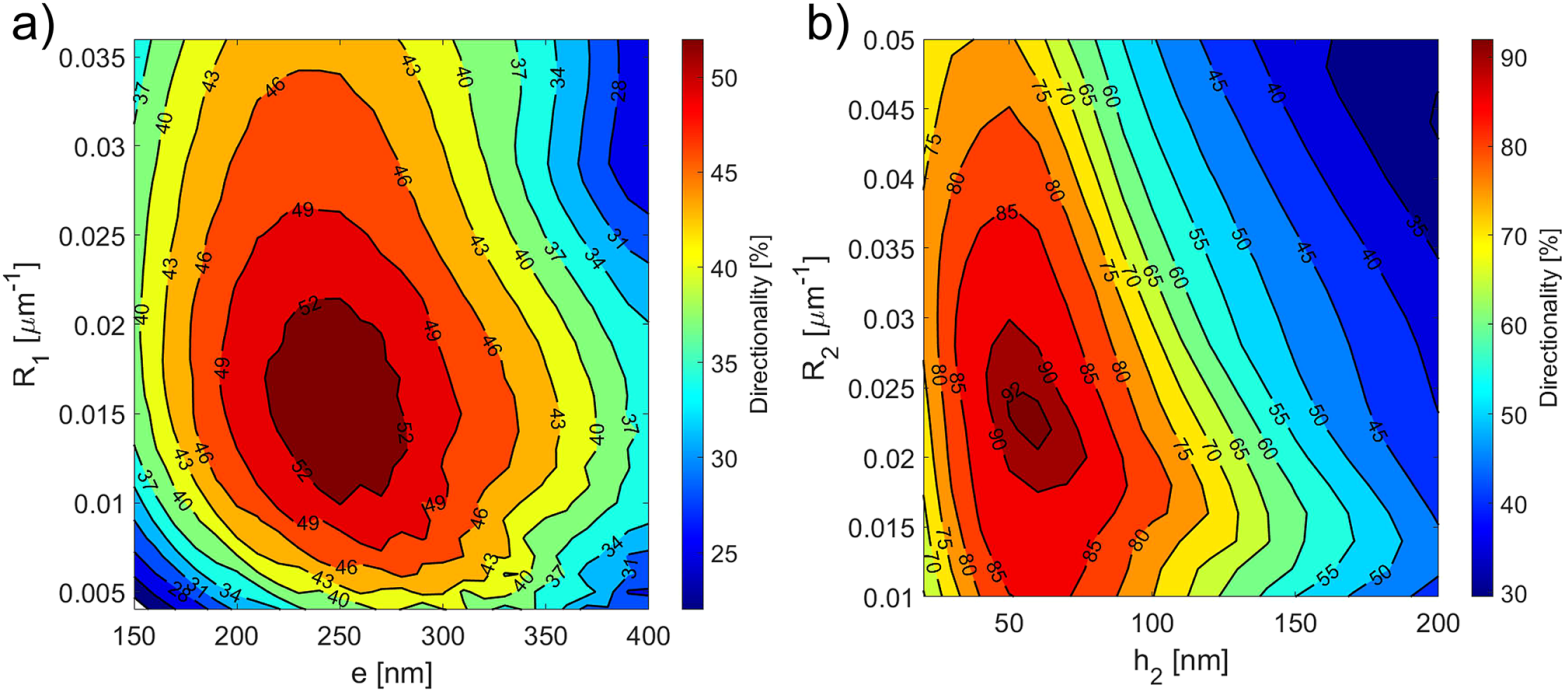
(**a**) Numerically simulated directionality at 1550 nm as a function of the etching depth *e* and bottom linear apodization factor $R_1$ considering a single level Si$_3$N$_4$ GC with a thickness of 400 nm (step 1 of the design); (**b**) numerically simulated directionality at 1550 nm as a function of the top layer thickness $h_2$ and top linear apodization factor $R_2$ for a dual level Si–Si$_3$N$_4$ GC with a Si$_3$N$_4$ thickness $h_1$ = 400 nm, *e* = 250 nm and $R_1$ = 0.016 $\upmu$m$^{-1}$ (step 2 of the design). For both simulations campaigns: *B* = 3 $\upmu$m, *T* = 1 $\upmu$m and *s* = 20 nm.

In the first step, the initial SiO$_2$ top cladding thickness *T* was set to 1 $\upmu$m. The GC was simulated as an out-coupling device by setting a TE$_{00}$-mode waveguide source in the Si$_3$N$_4$ waveguide placed at a distance of 12 $\upmu$m from the start of the GC, and a power monitor above the grating to record its directionality (see Fig. 2b for the simulation layout). Full vectorial 2D-FDTD simulations using FDTD Solutions$^{TM}$ were carried out by sweeping over the etching depth *e*, the bottom linear apodization factor $R_1$ and the grating period $\Lambda$. By considering the period $\Lambda$ for which the greatest value of directionality was achieved for each set of values of the *e* and $R_1$ parameters, the graph shown in Fig. 3a was obtained. As can be observed in the figure, a large region giving a directionality value greater than 52% can be identified. The best results from step 1 (*e* = 250 nm and $R_1$ = 0.016 $\upmu$m$^{-1}$) were fed into step 2, in which a top Si layer was considered at an initial minimum distance *s* = 20 nm (see Fig. 2c for the simulation layout). In this step, the peak directionality at $\lambda =$1550 nm was evaluated by sweeping over the Si thickness $h_2$, the top linear apodization factor $R_2$ and the grating period $\Lambda$, with the results reported in Fig. 3b. The addition of the top level significantly boosted the GC directionality, which reached a value as high as 92% for $h_2$ ranging between 50 and 65 nm, a $\Lambda$ of around 940 nm and an emission angle $\theta$ close to the vertical direction ($\theta$ in the range 2$^\circ$–3$^\circ$ depending on $h_2$).

**Figure 4:**
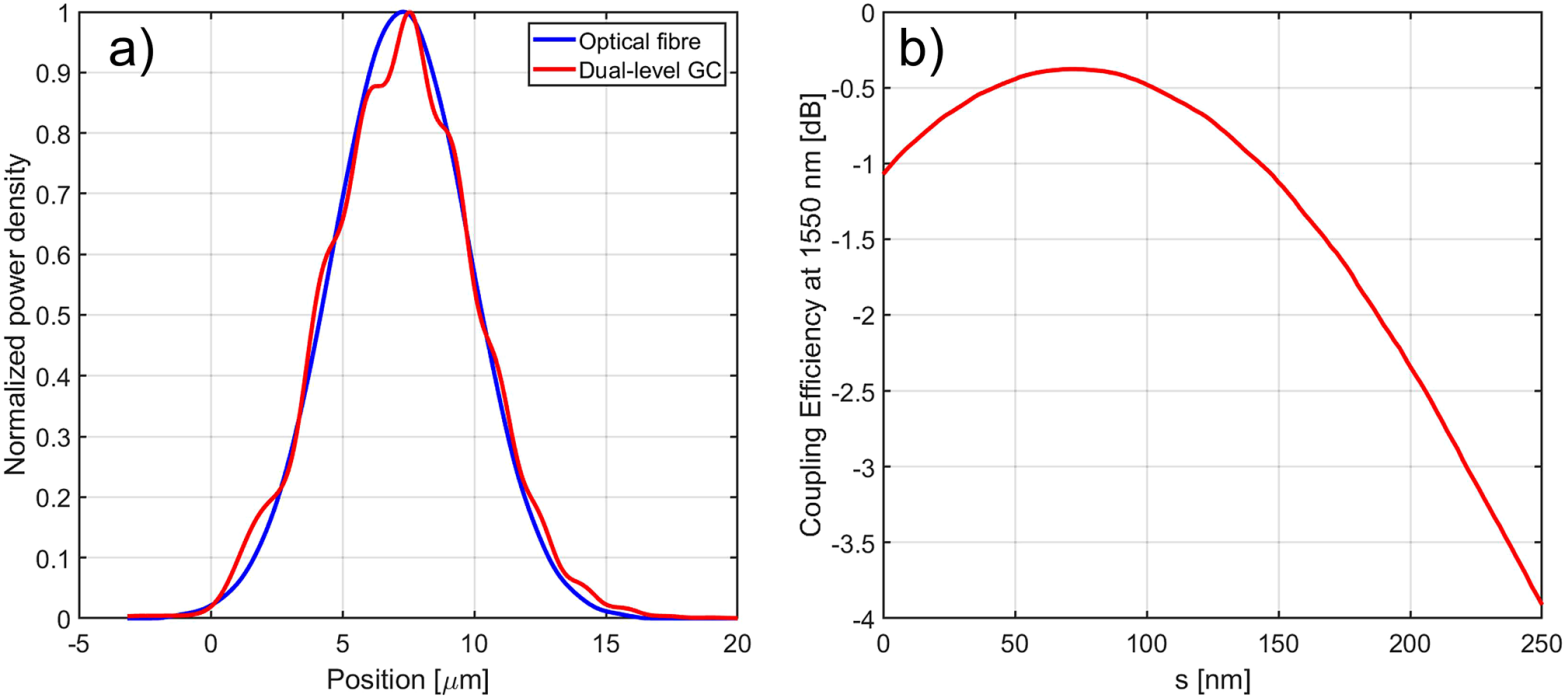
(**a**) Comparison of the normalized power density profiles of the optical fibre mode and of the diffracted mode from the dual-level Si–Si$_3$N$_4$ GC with 400 nm thick Si$_3$N$_4$ waveguide; (**b**) numerically simulated CE at 1550 nm as a function of the separation *s* between the two layers for the dual-level Si–Si$_3$N$_4$ GC with 400 nm thick Si$_3$N$_4$ waveguide. All the parameters used for these simulations are listed in Table 2 (table row with $\theta$ = 2$^\circ$).

**Figure 5:**
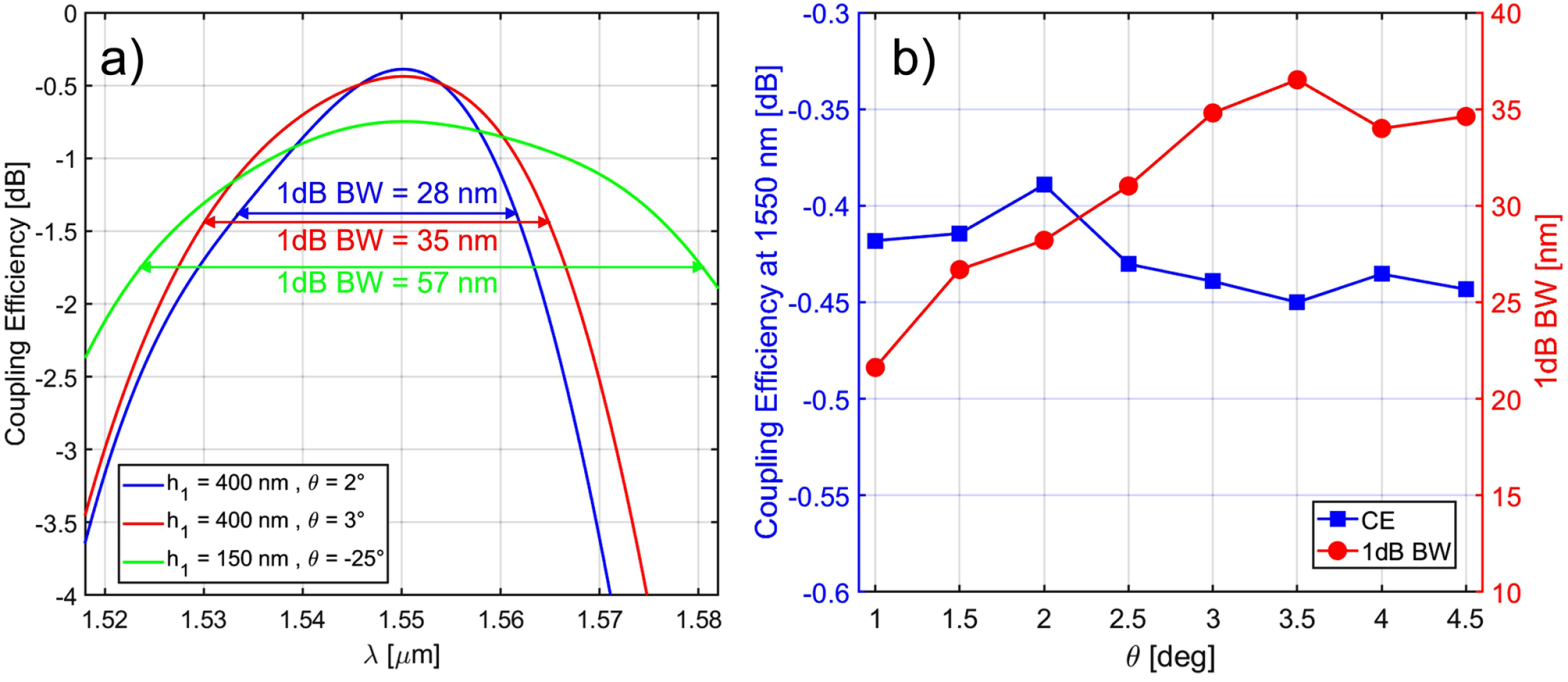
(**a**) Numerically simulated CE as a function of wavelength for dual-level Si–Si$_3$N$_4$ GCs considering 400 nm and 150 nm thick Si$_3$N$_4$ platforms; (**b**) peak CE at 1550 nm and 1 dB BW as a function of the coupling angle $\theta$ for dual-level Si–Si$_3$N$_4$ GC for a 400 nm thick Si$_3$N$_4$ platform.

In the third step, a particle swarm optimization algorithm was applied to the free parameter set {$e, h_2, R_1, R_2, \Lambda , s, T, \theta , z_f$} to optimize the CE starting from the best values found in the previous two steps. The CE was calculated by considering the grating as an in-coupling device, i.e. coupling light from a single mode fibre into the Si$_3$N$_4$ waveguide by means of the dual-level GC (see Fig. 2d for the simulation layout). The details of the simulation model can be found in the Methods section. The results of the optimization process for the Si–Si$_3$N$_4$ GC with a Si$_3$N$_4$ thickness of 400 nm are reported in Table 2 (table row with $\theta$ = 2$^\circ$), with the calculated CE versus wavelength shown in Fig. 5a. While the parameters *e*, $h_2$, $\Lambda$ and $\theta$ are very close to the results achieved from the directionality study in steps 1 and 2, the linear apodization factors $R_1$ and $R_2$ are larger, and this results from the CE optimization, which takes into account also the overlap between the field scattered by the GC and the optical fibre power profile. As can be seen from Fig. 4a, the employed dual-level apodization allows achieving a good overlap between the power density profiles of the optical fibre mode and the mode diffracted by the GC. A peak CE at 1550 nm and a 1 dB bandwidth (BW) of − 0.39 dB and 28 nm were found, respectively. A full vectorial 3D-FDTD simulation was also carried out on the optimized structure to take into account the GC width and verify the device performance. Considering a GC width equal to 14 $\upmu$m, a peak CE at 1550 nm of − 0.44 dB (0.05 dB lower than the value simulated in the 2D-case) and a 1 dB BW of 28 nm were found. The impact of the separation *s* between the two layers on the CE was then investigated, since this may represent a critical parameter for the fabrication process. Figure 4b shows that the final design is well tolerant to deviations of *s* from its nominal value: for a variation of ± 30 nm around the nominal value of 75 nm, a maximum CE decrease of around 0.14 dB can be observed. If the spacing between the two layers is increased beyond 150 nm, the CE starts to significantly decrease, since the device does not behave any longer like a single GC with a combination of two layers of teeth. The optimization procedure was then repeated considering different fixed fibre coupling angles and the results are shown in Fig. 5b. As can be seen, by selecting a wider angle, it is possible to increase the 1 dB BW of the GC at the expense of a small decrease in CE. For example, by selecting a coupling angle equal to 3$^\circ$, a 1 dB BW of 35 nm, covering the entire C band, can be achieved. This conclusion is in agreement with previous studies showing that, in general, the BW of a GC increases weakly with the coupling angle^27^.

**Table 2:** Design parameters and numerical results of the simulated Si–Si$_3$N$_4$ GCs for 500, 400, 300 and 150 nm thick Si$_3$N$_4$ photonic platforms.

Start parameters		Particle swarm optimization results									Performance	
$h_{1}$ (nm)	*B* ($\upmu$m)	*e* (nm)	$h_2$ (nm)	$R_1$ ($\upmu$m$^{-1}$)	$R_2$ ($\upmu$m$^{-1}$)	$\Lambda$ (nm)	*s* (nm)	*T* ($\upmu$m)	$\theta$	$z_f$ ($\upmu$m)	$CE_{peak}$	$BW_{1dB}$
500	3	295	62	0.0310	0.0385	914	20	1	2	7.6	− 0.39 dB	21 nm
400	3	240	65	0.0385	0.0405	941	75	0.46	2	7.2	− 0.39 dB	28 nm
400	3	255	65	0.0320	0.0350	948	40	0.4	3	7.4	− 0.44 dB	35 nm
300	3	169	74	0.0270	0.0275	976	78	0.35	4	7.4	− 0.7 dB	31 nm
150	6	150	81	0.0550	0.0220	792	215	0.85	− 25	5.1	− 0.75 dB	57 nm

In order to show the applicability of the proposed approach to other configurations, the same simulation campaign was carried out for three other Si$_3$N$_4$ waveguide thicknesses, namely 150, 300 and 500 nm. The results of the GC optimization procedure are reported in Table 2 and for all the considered Si$_3$N$_4$ platforms, CEs exceeding − 0.75 dB were numerically demonstrated, with a peak CE of − 0.39 dB simulated for the 500 nm case. Among these, the 150 nm thick Si$_3$N$_4$ platform has recently attracted significant attention thanks to its low propagation losses and is offered via dedicated runs as well as via open-access Multiproject Wafer (MPW) runs by several foundries, such as Ligentec (AN150 platform) and LioniX (TriPleX Si$_3$N$_4$-based platform)^40^. From the particle swarm optimization results, it can be seen that the trenches of the bottom Si$_3$N$_4$ layer are fully etched, which allows maximizing the scattering strength of the individual periods of the grating. For this particularly small Si$_3$N$_4$ thickness, a lower etching depth *e* would result in a smaller difference between the effective indices of the un-etched and etched regions, preventing the radiation of all the power by the GC in a given optical fibre mode diameter. A negative coupling angle of − 25$^\circ$ was found to maximize the CE for the initial choice of the BOX thickness, whose value was set equal to 6 $\upmu$m to minimize the power leakage to the substrate. A longer distance between the two GC layers was found to be optimal in this case, and this is due to the optical mode in the 150 nm thick Si$_3$N$_4$ waveguide being less confined in the core compared to the other reported cases. A peak CE and 1 dB BW of − 0.75 dB and 57 nm, respectively, were numerically demonstrated.

As a final consideration on the proposed design approach, it should be noted that a fixed BOX thickness was considered, with the coupling angle $\theta$ as a free parameter. The optimization algorithm allows determining the best angle for which constructive interference occurs between the light reflected at the BOX-Si substrate interface and the upper diffracted light from the GC. The same design methodology can be carried out in the analogous case where a specific coupling angle $\theta$ is required, with the BOX thickness as a free parameter to be optimized.

### Dual-level Si$_x$N$_y$–Si$_3$N$_4$ grating couplers

**Figure 6:**
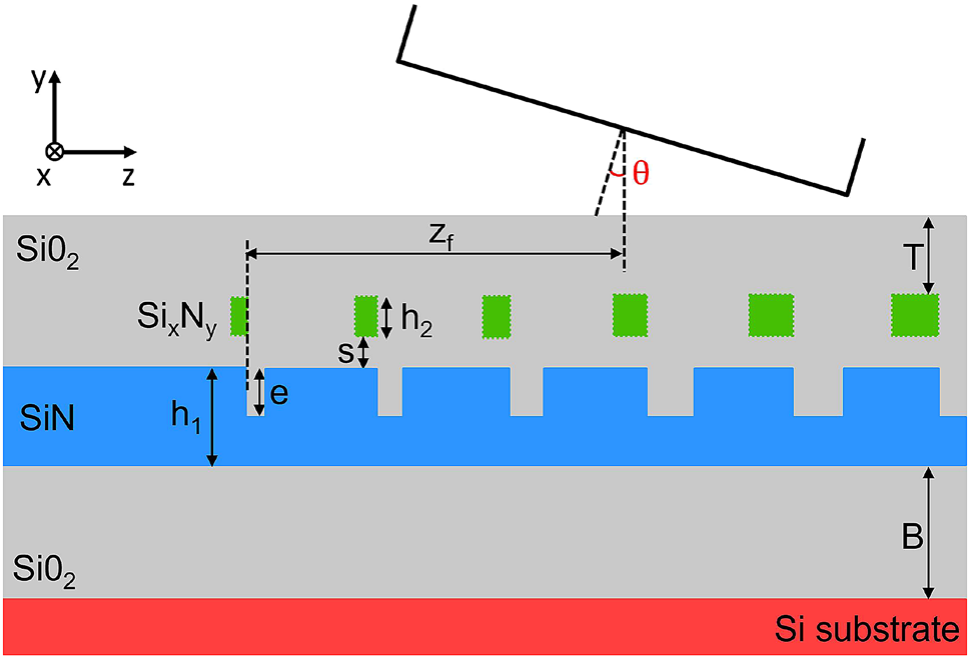
2D schematic view of the proposed dual-level Si$_x$N$_y$–Si$_3$N$_4$ GC.

The performance of dual-level GCs employing a different material in the top layer was considered next. Specifically, Si-rich silicon nitride (Si$_x$N$_y$, 2.1 $\le$ $n_2$ $\le$ 3.3) and stoichiometric Si$_3$N$_4$ were considered as top layer materials in place of Si. The layout of the simulated dual-level Si$_x$N$_y$–Si$_3$N$_4$ GC is shown in Fig. 6.

**Figure 7:**
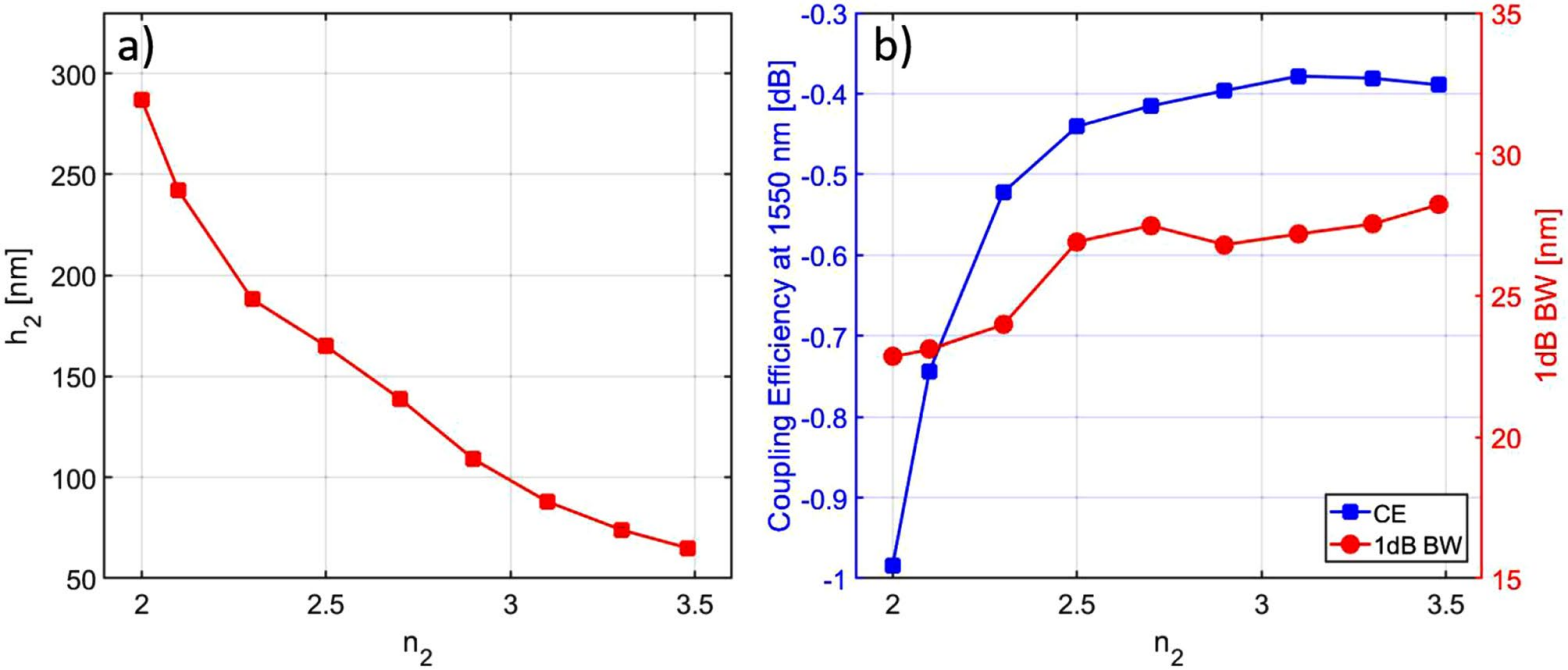
(**a**) Thickness $h_2$ which maximizes the CE as a function of the top level refractive index $n_2$; (**b**) peak CE at 1550 nm and 1 dB BW as a function of $n_2$ for dual-level Si$_x$N$_y$–Si$_3$N$_4$ GC for a 400 nm thick Si$_3$N$_4$ platform.

The optimization procedure was carried out for discrete values of the refractive index and the final particle swarm optimization results are reported in Table 3. Figure 7a shows the top level thickness $h_2$ which maximizes the CE for each choice of refractive index value $n_2$ for the top layer, while Fig. 7b shows the numerically simulated peak CE at 1550 nm and the corresponding 1 dB BW for each of these values of $h_2$. As may be expected, when decreasing $n_2$, a thicker Si$_x$N$_y$ top layer is required to achieve the best performance in terms of CE. From Fig. 7b it can be seen that the use of $n_2$ values ranging between 2.7 and 3.48 yields similar results, with CE > 90% and 1 dB BW around 27 nm, while a further decrease in the refractive index resulted in a gradual degradation in performance.

**Table 3:** Design parameters of the simulated Si$_x$N$_y$–Si$_3$N$_4$ GCs for a 400 nm thick Si$_3$N$_4$ photonic platform with Si$_x$N$_y$ and Si$_3$N$_4$ as top layer materials.

Initial parameters			Particle swarm optimization results								
$h_{1}$ (nm)	$n_2$	*B* ($\upmu$m)	*e* (nm)	$h_2$ (nm)	$R_1$ ($\upmu$m$^{-1}$)	$R_2$ ($\upmu$m$^{-1}$)	$\Lambda$ (nm)	*s* (nm)	*T* [$\upmu$m]	$\theta$	$z_f$ ($\upmu$m)
400	3.3	3	245	74	0.0395	0.0405	942	67	0.48	1.8	7.2
400	3.1	3	245	88	0.0395	0.0415	941	57	0.48	1.8	7.2
400	2.9	3	245	109	0.0390	0.0410	941	48	0.47	1.7	7.3
400	2.7	3	240	139	0.0440	0.0400	941	32	0.47	1.7	7.1
400	2.5	3	240	165	0.0410	0.0420	940	20	0.47	1.7	7.1
400	2.3	3	230	188	0.0345	0.0375	937	20	0.45	1.3	8
400	2.1	3	230	242	0.0295	0.0380	938	20	0.37	1.3	8.3
400	2	3	230	287	0.0295	0.0375	939	20	0.31	1.3	8.3

**Figure 8:**
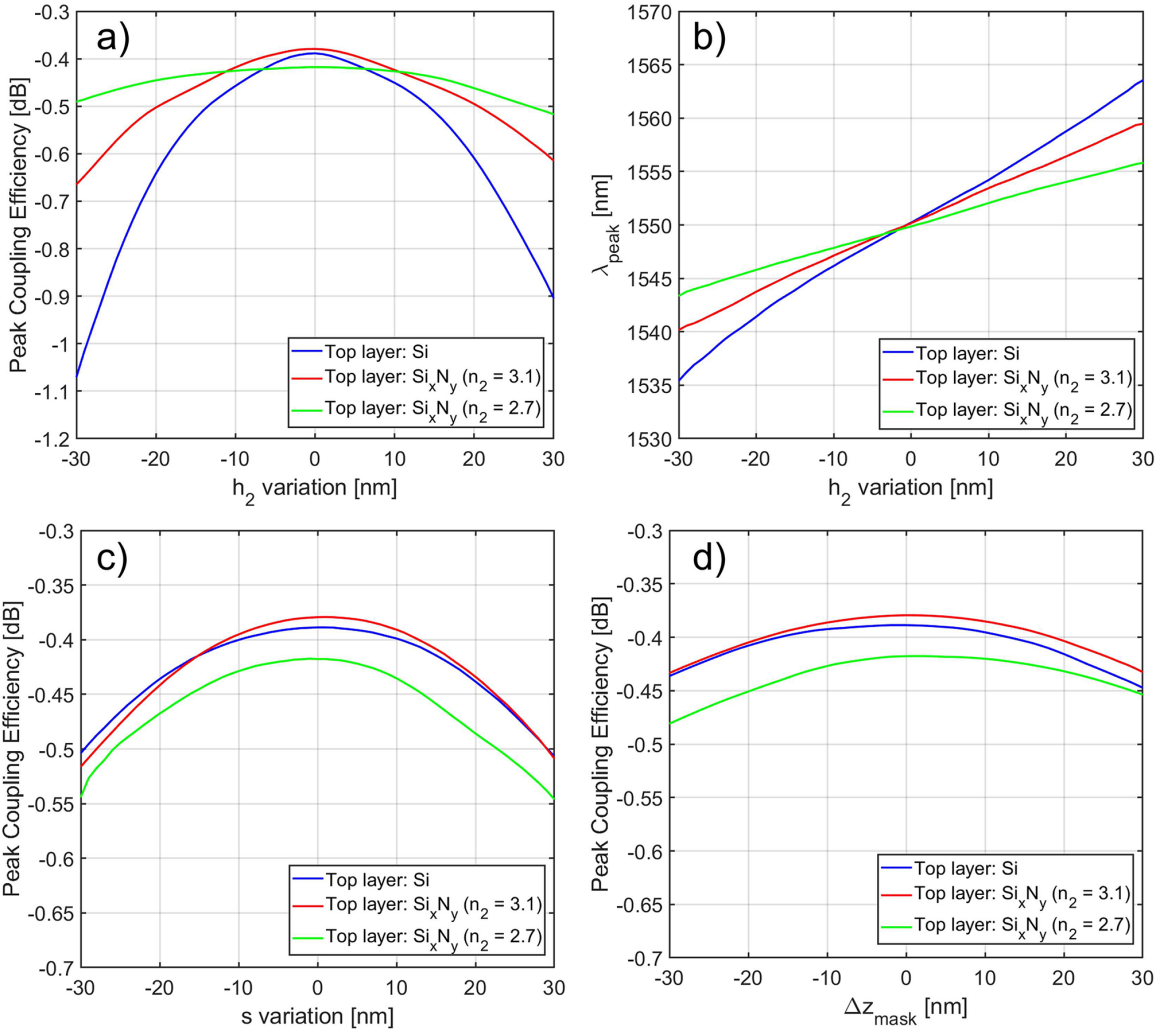
Grating sensitivity to critical fabrication parameters: peak CE dependence on (**a**) top thickness $h_2$, (**c**) distance between the two layers *s* and (**d**) layers misalignment $\Delta z_{mask}$ variation; (**b**) peak wavelength dependence on $h_2$ variation.

The GC sensitivity to fabrication errors was also evaluated. In particular, the tolerance of the GC performance to deviations in the fabrication due to the addition of the top layer was considered. Figure 8 shows the impact of $h_2$, *s* and $\Delta z_{mask}$ variations with respect to the nominal values on the GC peak CE, where $\Delta z_{mask}$ accounts for the offset between the two layers along the $\hat{z}$ direction. Considering the $h_2$ parameter, Fig. 8a suggests that the use of a top material with a lower refractive index leads to a more robust design in terms of the peak CE. Specifically, a CE degradation of less than 0.1 dB was observed for a ± 30 nm $h_2$ variation when a Si$_x$N$_y$ top layer with $n_2$ = 2.7 was considered, whereas a CE decrease of 0.7 dB was observed for the same $h_2$ variation interval when Si was employed as top level material. Similarly, the choice of lower $n_2$ values resulted in a lower peak wavelength shift from the designed 1550 nm value, as shown in Fig. 8b. With regard to the remaining two parameters, the simulations showed that the choice of $n_2$ did not have a significant impact on the fabrication tolerance, as can be observed in Fig. 8c,d. A CE decrease of around 0.13 dB and 0.06 dB with a maximum peak wavelength shift of 0.6 nm and 3.4 nm could be observed for a ± 30 nm variation in the *s* and $\Delta z_{mask}$ parameters, respectively.

## Discussion and conclusions

A new dual-level GC design methodology that can be applied to an arbitrary Si$_3$N$_4$ photonic platform with the use of different top layer materials was presented. The GC layout consisted of two GC layers separated by a small SiO$_2$ thickness of a few tens of nm, with a bottom Si$_3$N$_4$ waveguide acting as the guiding layer. The fill-factors of both GCs were linearly apodized, with opposite apodization signs: the width of the bottom teeth was decreasing when moving away from the waveguide, whereas the width of the top teeth was increasing. The use of two inverse linear apodizations allowed the mode mismatch at the GC interface to be minimized and reduced the set of simulation parameters. Unlike previously reported designs^13^, instead of optimizing each GC period and fill-factor independently, the GC teeth and trenches of both layers were defined here by using only three parameters, i.e. the two linear apodization factors and the common grating period. This allowed a large parameter space and several possible GC configurations to be explored, while considering also different etching depths for the bottom Si$_3$N$_4$ layer, separation distances between the two layers and top GC thicknesses. The first two steps of the design procedure consisted in sweeping the bottom and, then, the top GC parameters respectively, to maximize directionality. The results from these parameter sweeps were fed into a particle swarm optimization algorithm, which optimized the GC CE by maximizing the overlap between the field scattered by the grating and the optical fibre power profile.

The GC optimization procedure was applied to different Si$_3$N$_4$ platforms, with 150, 300, 400 and 500 nm waveguide thicknesses, considering initially Si as the top level material. The 400 nm thick Si$_3$N$_4$ platform is widely used to realize passive photonic interconnects, while the 150 nm thick Si$_3$N$_4$ platform is attracting considerable interest thanks to the possibility of achieving low propagation losses, since the optical mode is mainly propagating in the surrounding SiO$_2$. Peak CEs of − 0.39 dB and − 0.75 dB were simulated for the 400 nm and 150 nm Si$_3$N$_4$ platform, respectively, which represent, to the best of our knowledge, the best numerical results ever achieved without the use of embedded back-reflectors. Finally, the use of Si$_x$N$_y$ (with 2.0 $\le$ $n_2$ $\le$ 3.3) as the top layer material was investigated. Si$_x$N$_y$ layers with a tunable refractive index can be deposited by varying the gas composition of the film forming reactants (SiH$_2$Cl$_2$, SiH$_4$ and NH$_3$, N$_2$ are commonly used for Si and N, respectively) injected into the reaction chamber^31^. In this way, the ratio of the Si and N content in the Si$_x$N$_y$ film can be varied, providing a further degree of freedom in dispersion and device engineering. Several compositions of Si$_x$N$_y$ have been reported in the literature, with a refractive index varying in the range 2.1–3.1^41–44^. Our simulations showed that the performance of the GCs was not affected by the choice of the top-layer material, as long as the refractive index remained in the range 2.7–3.48, in which case, a simulated CE > − 0.45 dB (90%) was predicted. However, this choice affected considerably the fabrication tolerances that could be achieved. At the expense of a slightly compromised CE, a top-layer material with a lower refractive index than Si resulted in designs that were significantly more robust in variations of the top-layer thickness. Finally, it is worth noting that the use of a dual/multi-level dual/multi-refractive index Si$_x$N$_y$–Si$_3$N$_4$ platform could be a better alternative to the hybrid Si$_3$N$_4$-SOI platform, thanks to its relaxed fabrication tolerances, refractive index tuning capabilities and layer deposition versatility.

## Methods

### Numerical simulations

All Full Vectorial FDTD numerical simulations were performed using FDTD Solutions$^{TM}$ (from Lumerical Inc.). The 2D computational area was set to be 36.5 $\upmu$m wide and 7 $\upmu$m (11 $\upmu$m) high for the 300, 400 and 500 nm (150 nm) thick Si$_3$N$_4$ platforms. For the 3D computational area, the simulation region was set to be 20 $\upmu$m large in the $\hat{x}$ direction (see Fig. 2d). For the refractive index of Si and SiO$_2$, the data reported by Palik^45^ were employed, resulting in $n_{Si}$ = 3.48 and $n_{SiO_2}$ = 1.44 at the central design wavelength of 1550 nm. Regarding the refractive index of the Si$_x$N$_y$ compositions (2.1 $\le$ $n_{Si_xN_y}$ $\le$ 3.3) and Si$_3$N$_4$ ($n_{Si_3N_4}$ = 2.0), a constant refractive index was considered in the wavelength simulation range (1.5 $\upmu$m - 1.6 $\upmu$m). The simulation mesh was defined using the conformal mesh method embedded in Lumerical, with the highest possible setting accuracy value of 8. An additional refined mesh, with a minimum feature size of 5 nm inside the material, was added in the GC region. A number of periods equal to 23 was considered for each grating design. For steps 1 and 2 of the design procedure (see Fig. 2b,c), the power diffracted upwards by the GC, which determined the GC directionality, was calculated using a frequency-domain power monitor, with the number of frequency points set equal to 500 in the wavelength range from 1.5 to 1.6 $\upmu$m. For step 3 of the design procedure (see Fig. 2d), the fibre source, which was modeled as a Gaussian beam with a mode field diameter (MFD) of 10.4 $\upmu$m and embedded in the air region above the top SiO$_2$ cladding, was tilted by an angle $\theta$ with respect to the vertical direction. The electric field of the beam was polarized along the $\hat{x}$ direction (see Fig. 2d), which resulted in the incoming light being coupled to the TE$_{00}$-mode of the Si$_3$N$_4$ waveguide. A frequency-domain power monitor, placed along the Si$_3$N$_4$ waveguide at a distance of 12 $\upmu$m from the start of the GC, was used to determine the amount of power coupled in the fundamental TE mode of the waveguide and, hence, the CE.
